# Targeting SIRT1 stability: arctiin ameliorates doxorubicin-induced cardiac injury by inhibiting SMURF2 binding and enhancing protective lipid metabolism

**DOI:** 10.3389/fphar.2026.1797815

**Published:** 2026-04-23

**Authors:** Teng Teng, Yujia Huo, Zhuoyu Shen, Haiyang Ni, Yupei Yuan, Sichi Xu, Nan Zhang, Qizhu Tang

**Affiliations:** 1 Department of Cardiology, Renmin Hospital of Wuhan University, Wuhan, China; 2 Hubei Key Laboratory of Metabolic and Chronic Diseases, Wuhan, China

**Keywords:** arctiin, doxorubicin-induced cardiotoxicity, lipid metabolism, SIRT1, SMURF2

## Abstract

**Background:**

Doxorubicin (DOX) is a highly effective chemotherapeutic agent, but its severe cardiotoxicity limits its clinical use. This study aimed to investigate whether the natural compound Arctiin can alleviate DOX-induced cardiomyopathy and its underlying mechanisms.

**Methods:**

DOX-treated H9C2 cardiomyocytes and mouse models were used. Cardiac function, myocardial injury, oxidative stress, endoplasmic reticulum (ER) stress, apoptosis, and lipid metabolism were assessed by echocardiography, histological staining, molecular biology assays, and lipidomics. SIRT1 knockdown/knockout models were employed to verify its necessity.

**Results:**

Arctiin significantly improved DOX-induced cardiac dysfunction and myocardial damage in mice. Mechanistically, Arctiin directly binds to the SIRT1 protein and disrupts its interaction with the E3 ubiquitin ligase SMURF2, thereby suppressing SIRT1 ubiquitination and subsequent proteasomal degradation, ultimately stabilizing and upregulating SIRT1 protein levels. The activated SIRT1 then upregulates the expression of its downstream effectors, NRF2 and HO-1, collectively alleviating oxidative stress and ER stress and inhibiting apoptosis in cardiomyocytes. Furthermore, lipidomics analysis revealed that Arctiin reversed DOX-induced cardiac lipid metabolic dysregulation in a SIRT1-dependent manner. However, all these protective effects of Arctiin were completely abolished in SIRT1-knockdown or knockout cell and mouse models.

**Conclusion:**

This study demonstrates that Arctiin targets the SIRT1 protein and inhibits its SMURF2-mediated ubiquitination and degradation, thereby stabilizing and activating the SIRT1/NRF2 pathway. This leads to improved lipid metabolism, reduced oxidative and ER stress, and ultimately protection against DOX-induced cardiotoxicity. These findings provide a novel theoretical basis for Arctiin as a potential cardioprotective agent.

## Graphical Abstract

**Figure:**
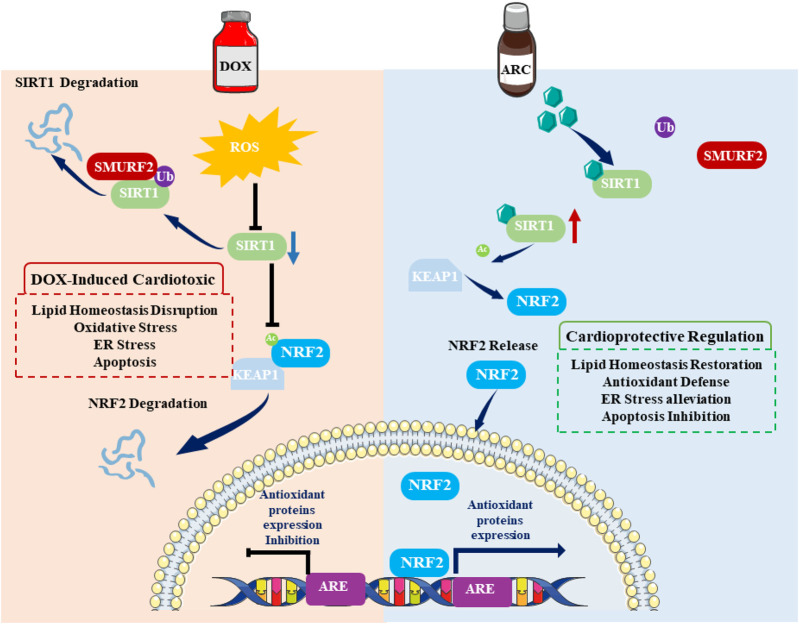
Doxorubicin (DOX) induces cardiotoxicity through a pathological cascade involving oxidative stress, activation of ER stress, excessive apoptosis, and dysregulation of lipid metabolism, ultimately leading to cardiomyocyte damage and cardiac dysfunction. Arctiin counteracts this process by directly binding to SIRT1 and inhibiting its SMURF2-mediated ubiquitination and proteasomal degradation, thereby stabilizing and upregulating SIRT1 protein levels. The accumulated SIRT1 then initiates a protective SIRT1/NRF2 pathway, which enhances antioxidant defense, alleviates ER stress, inhibits apoptosis, and restores lipid homeostasis. The absence of SIRT1 abolishes these protective effects, confirming its role as the centsral mediator. Thus, by preventing SIRT1 degradation, Arctiin disrupts the toxic cycle driven by DOX, offering a promising therapeutic strategy for chemotherapy-associated cardiac injury.

## Introduction

Doxorubicin (DOX), is a kind of anthracycline antibiotic discovered earlier, which is used as a broad-spectrum chemotherapeutic drug. Even to this day, there is still a relatively large amount use as an effective antitumor drug for treating several adult and pediatric malignancies, such as breast cancer, Hodgkin’s disease, lymphoblastic leukemia, etc., (Kalyanaraman, 2020; Carvalho et al., 2014). However, the most barrier in clinical application is the existence of elicitation of organ toxicity especially related to cardiotoxicity. The side effects, especially cardiotoxicity, occur at any stage of treatment. According to its different severity and clinical manifestation, cardiotoxicity can be roughly classified into the following three categories (Kalyanaraman, 2020): acute cardiotoxicity (Carvalho et al., 2014); early-onset chronic (subacute) cardiotoxicity, and (Cardinale et al., 2015) late-onset chronic cardiotoxicity (Cardinale et al., 2015). The incidence of acute cardiotoxicity in cancer patients treated with DOX is approximately 30%, 11% of patients among these patients can be controlled and are usually reversible if they were treated immediately as soon as the side effect initiation (Ferrans et al., 1997; Lefrak et al., 1973). Unlike acute cardiotoxicity, subacute cardiotoxicity is uncommon. Subacute cardiotoxicity is usually observed several days even weeks after the last administration of DOX, mainly including pericarditis and myocarditis (Yu et al., 2018). Chronic cardiotoxicity is related to progressive myocardial dysfunction, which develops years even decades after the end administration of DOX, the cumulative dosage of DOX is the important risk factor of the incidence of congestive heart failure (CHF) (Yu et al., 2018). The toxicity can lead to dilated cardiomyopathy and CHF (Ewer and Ewer, 2010). Until now, there isn’t an effective therapy that can eliminate the cardiotoxicity of DOX, preventing today’s cancer patients from becoming tomorrow’s heart patients.

The mechanism of DOX-induced cardiomyopathy involves a lot of aspects, the generation of excessive reactive oxygen species (ROS) plays a pivotal role in DOX-induced cardiotoxicity. Mitochondria are the most easily and severely damaged subcellular organelle in DOX-induced cardiotoxicity (Wenningmann et al., 2019). The accumulation of DOX in mitochondria facilitates the production of ROS and reactive nitrogen species (Priya et al., 2017; Weinstein et al., 2000), these reactive in turn enhance the degree of oxidative stress. In addition, the endoplasmic reticulum (ER) is also a component of redox balance. The ER redox state depends on the ER protein-folding homeostasis, the accumulation of unfolded protein due to disulfide bond formation disorders causes ER stress. Many views support that ER stress and oxidative stress accentuate each other in a positive feed-forward loop, ultimately activating pro-apoptosis signaling (Cao and Kaufman, 2014; Malhotra and Kaufman, 2007).

Importantly, dysregulated lipid metabolism has emerged as a critical pathological process that intersects with and amplifies these key mechanisms. Toxic lipid accumulation, such as ceramides and saturated fatty acids, directly fuels mitochondrial ROS production and lipid peroxidation, exacerbating oxidative stress (Kretzschmar et al., 2021). Furthermore, specific lipid species can activate pro-apoptotic pathways independently or synergistically with ER stress signals. Conversely, oxidative and ER stress disrupt cellular lipid homeostasis, creating a vicious cycle that drives cardiomyocyte death and dysfunction (Arunachalam et al., 2013). Thus, lipid metabolic imbalance is not merely a consequence but an integral driver within the interconnected network of DOX-induced cardiotoxicity.

SIRT1, a NAD^+^-dependant deacetylase, and the critical antioxidative gene has been verified to play a potent role in DOX-induced cardiotoxicity. SIRT1 is involved in many mechanisms about DOX-induced cardiotoxicity, such as oxidative stress, mitochondria dysfunction, ER stress, apoptosis, inflammatory response, lipid metabolism, and other pathologic processes (Wang et al., 2021; Domi and Hoxha, 2025). Nuclear factor erythroid 2-related factor 2 (NRF2) is also a part of the antioxidative system which is regulated by Sirt1 deacetylated modified. NRF2 binds to Kelch-like ECH-associated protein 1 (KEAP1) and is localized in the cytoplasm under physiological conditions. Once stimulated in stressed conditions, the NRF2 dissociated from KEAP1, which then binds to antioxidant response elements (ARE), and upregulates downstream target genes (Kura et al., 2020; Chen et al., 2015).

Arctiin, existed in many species of Asteraceae and first isolated from Arctium lappa, is a kind of glucoside of artigenin and possesses several pharmacological effects including anti-viral, anti-inflammatory, anti-proliferative, and anticancer properties; besides, it was reported to play a part of platelet-activating factor antagonist and calcium antagonist (Rolnik and Olas, 2021). Moreover, arctiin could alleviate intracellular ROS generation caused by H_2_O_2_ (Rolnik and Olas, 2021). Our previous research indicated that arctiin attenuated cardiac hypertrophy, and suppressed cardiac fibrosis (Li et al., 2017). Whether arctiin plays an antioxidant role in DOX-induced cardiotoxicity is unclear. Therefore, this study aims to investigate the effect of arctiin on DOX-induced cardiotoxicity and elucidate the potential mechanisms.

## Results

### Arctiin improved DOX-induced cardiac function in mice

**FIGURE 1:**
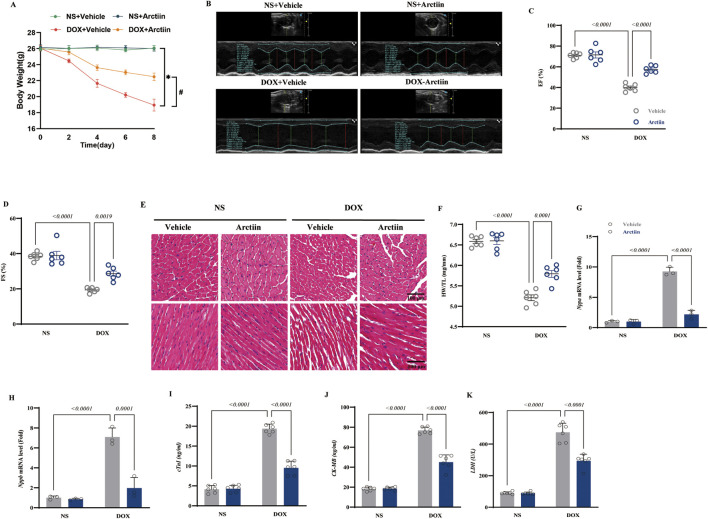
Arctiin prevented deterioration of cardiac function from DOX-induced injury. **(A)** Changes in body weight of four groups (n = 6). **(B–D)** Cardiac echocardiographic examination and analysis (n = 6). **(E,F)** H&E staining of heart and ratio of HW/TL on 7th day after exposure of DOX (n = 6). **(G,H)** mRNA level of *Nppa* and *Nppb* of four groups (n = 3). **(I-K)** Contents of cTnI, CK-MB, and LDH in serum of four groups (n = 6). Data were presented as mean ±S.D.

Mice in the experimental group received a single intraperitoneal injection of DOX to establish a model of DOX-induced heart damage. As shown in the results, DOX treatment induced significant weight loss in mice (Figure 1A). Cardiac function was subsequently assessed by echocardiography. DOX administration adversely affected both the ejection fraction (EF) and left ventricular fractional shortening (LVFS), impairments that were ameliorated by Arctiin treatment (Figures 1B–D). Consistent with the changes in body weight, the ratio of heart weight to tibia length was also reduced in the DOX group, and these losses in both body weight and heart mass were alleviated by Arctiin. Histological analysis with HE staining revealed that DOX induced cardiomyocyte atrophy and a degree of myofibril disarray, which were mitigated by Arctiin (Figures 1E,F). Furthermore, the mRNA levels of cardiac stress markers *Nppa* and *Nppb* were elevated in DOX-treated mice compared to those receiving Arctiin intervention (Figures 1G,H). Finally, we examined sensitive plasma biomarkers of myocardial injury, including cardiac troponin I (cTnI), Creatine Kinase-MB Form (CK-MB), and lactate dehydrogenase (LDH). These biomarkers were significantly elevated following DOX treatment, and Arctiin administration alleviated this chemotherapy-induced cardiac injury, as evidenced by the reduction of these injury markers in the blood (Figures 1I–K).

### Arctiin ameliorated oxidative stress in DOX-induced cardiotoxicity *in vivo*

**FIGURE 2:**
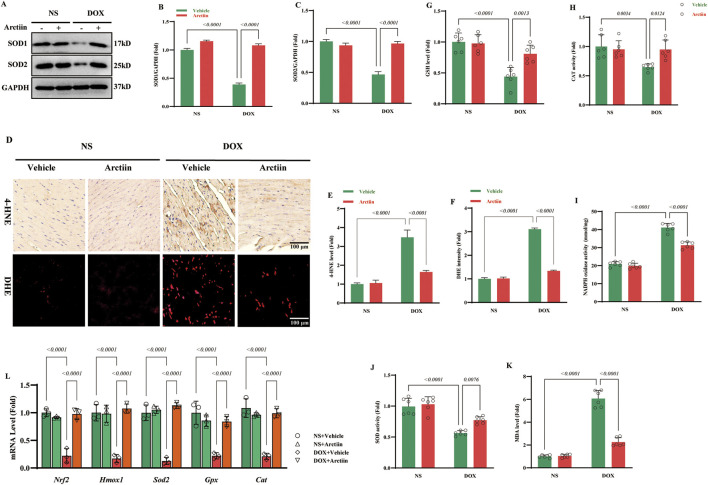
Arctiin alleviated oxidative injury lured by DOX in mice. **(A–C)** Representative western blot images and statistical results (n = 3). **(D–F)** D represents 4-HNE and DHE staining of hearts, E and F represent statistical results of of two kinds of staining respectively (n = 3). **(G–K)** The contents of GSH level, CAT activity, NADPH oxidase activity, SOD activity, and MDA in hearts (n = 6). **(L)** Representative of mRNA level of oxidative stress related genes (n = 3). Data were presented as mean ±S.D.

Oxidative stress is a kernel mechanism of DOX-induced cardiotoxicity, which may cause ER stress and eventually results in massive cardiomyocytes apoptosis (Wang et al., 2021). To investigate the role of Arctiin in mitigating oxidative stress *in vivo*, we first examined key antioxidant proteins. Western blot analysis confirmed the protective effect of Arctiin, as the DOX-induced reduction in protein levels of Superoxide dismutase 1 (SOD1) and SOD2 was restored by Arctiin administration (Figures 2A–C). Furthermore, 4-Hydroxynonenal (4-HNE) immunostaining and DHE fluorescence staining revealed that Arctiin treatment significantly attenuated the DOX-induced accumulation of lipid peroxidation product (4-HNE) and the overproduction of ROS in cardiac tissue (Figures 2D–F). To further characterize the oxidative stress status, we assessed a panel of biochemical markers. The results showed that Arctiin treatment effectively reversed the DOX-induced elevations in myocardial Malondialdehyde (MDA) content and nicotinamide adenine dinucleotide phosphate (NADPH) oxidase activity. Concurrently, it restored the decreased activities of SOD and Catalase (CAT), as well as the level of glutathione (GSH) (Figures 2G–K). Consistent with these findings, the mRNA levels of key antioxidant genes related to the antioxidant response element (ARE) were upregulated in the Arctiin-treated group (Figure 2L).

### Arctiin attenuated endoplasmic reticulum stress *in vivo*

**FIGURE 3:**
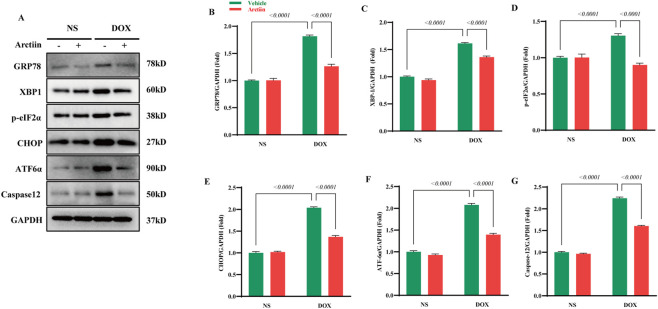
Arctiin suppressed endoplasmic reticulum stress after DOX hit in mice. **(A)** Representative western blot images and **(B–G)** statistical results of proteins related to ER stress, including Glucose-regulated protein-78 (GRP78), X-box binding protein-1 (XBP1), Phosphorylated-eukaryotic translation initiation factor-2 (p-eIF2), C/EBP homologous protein (CHOP), Activating transcription factor 6 (ATF6), and Caspase-12 (n = 3). Data were presented as mean ±S.D.

Previous studies demonstrated that ER stress played a key role in the development of DOX-induced cardiotoxicity (Yu et al., 2018). Thus, we investigated whether Arctiin could alleviate DOX-induced endoplasmic reticulum (ER) stress. Under ER stress conditions, the Unfolded Protein Response (UPR) is activated. This involves the dissociation of the chaperone Glucose-Regulated Protein 78 (GRP78) from UPR sensors, which then become activated. While the UPR initially aims to enhance the protein-folding capacity of the ER, sustained stress leads to the upregulation of GRP78 expression (Pfaffenbach and Lee, 2011). Our results showed that the protein level of GRP78 was significantly upregulated in DOX-treated mice, and this increase was attenuated by Arctiin administration. The protective effect of Arctiin against DOX-induced cardiotoxicity was further supported by its modulation of other key UPR signaling branches. The activation levels of sensors including p-eIF2α, XBP1, and ATF6α were elevated following DOX treatment, and Arctiin administration effectively reduced this activation. Furthermore, we found that the expression of important ER stress-mediated pro-apoptotic markers, CHOP and Caspase-12, was increased by DOX, and this effect was counteracted by Arctiin treatment (Figures 3A–G).

### Arctiin prevented cardiomyocytes from apoptosis *in vivo*

**FIGURE 4:**
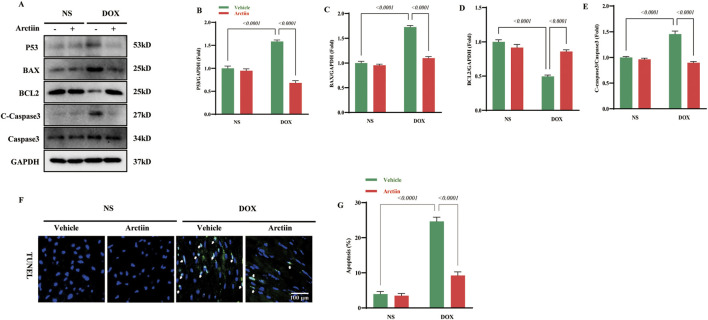
Arctiin attenuated DOX-induced cardiomyocyte apoptosis in mice. **(A–E)** Representative western blot images and Statistical results of proteins related to apoptosis, including P53, BAX, BCL2, and c-Caspase3 (n = 3). **(F)** Represent TUNEL staining and **(G)** quantitative statistical result (n = 3). White arrows represent TUNEL-positive cells. Data were presented as mean ±S.D.

A lot of evidence demonstrated that cardiomyocyte apoptosis is a common pathological feature of acute and chronic side effect in DOX therapy (Wang et al., 2021). Western blot analysis revealed that DOX treatment significantly increased the expression of the pro-apoptotic proteins p53 and BAX, while decreasing the expression of the anti-apoptotic protein BCL2 (Figures 4A–D). Consistent with this, the level of the apoptosis executor cleaved-caspase 3 was also elevated in the DOX group, and all these changes were effectively reversed by Arctiin administration (Figure 4E). Furthermore, TUNEL staining results confirmed that DOX treatment induced marked cardiomyocyte apoptosis, which was significantly attenuated by Arctiin (Figures 4F,G). These findings collectively demonstrate that Arctiin ameliorates DOX-induced cardiomyocyte apoptosis, aligning with established evidence that apoptosis is a central pathological feature of DOX-induced cardiotoxicity.

### Arctiin protected cardiomyocytes from DOX insult *in vitro*

**FIGURE 5:**
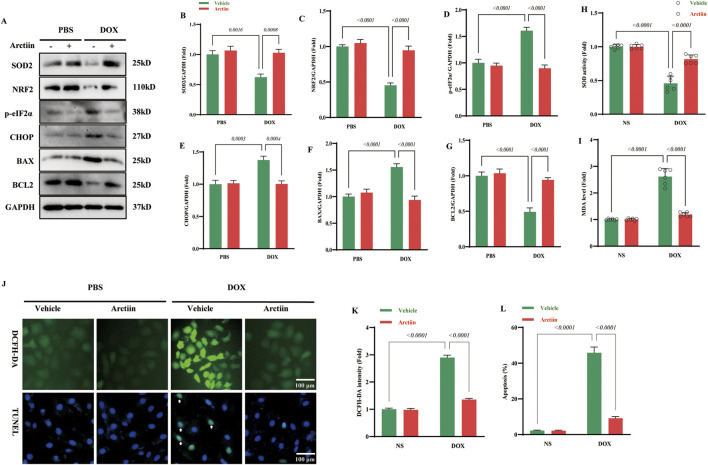
Arctiin could attenuate DOX-induced cardiomyocytes injury *in vitro*. **(A)** Representative western blot images and **(B–G)** statistical results of proteins related to oxidative stress, endoplasmic reticulum stress, and apoptosis (n = 3). **(H,I)** The contents of SOD activity and MDA in hearts (n = 6). **(J–L)** Represent DCFH-DA staining, TUNEL staining and their quantitative statistical results (n = 3). White arrows represent TUNEL-positive cells. Data were presented as mean ±S.D.

Next, we investigated the protective effect of Arctiin *in vitro*. Consistent with the *in vivo* findings, Arctiin demonstrated a potent inhibitory effect on oxidative stress. Western blot analysis confirmed the multi-faceted protective mechanisms of Arctiin at the molecular level. Specifically, Arctiin enhanced the cellular antioxidant defense, as indicated by the upregulation of key antioxidant proteins SOD2 and NRF2. Concurrently, it alleviated DOX-induced endoplasmic reticulum (ER) stress, evidenced by the downregulation of activated UPR markers such as p-eIF2α and the pro-apoptotic transcription factor CHOP. Furthermore, Arctiin exhibited a pronounced anti-apoptotic effect by decreasing the expression of the pro-apoptotic protein BAX while elevating the level of the anti-apoptotic protein BCL-2 (Figures 5A–G). These molecular improvements translated into significant functional benefits. At the biochemical level, Arctiin increased SOD activity and reduced MDA content (Figures 5H,I). In addition, DCFH-DA staining and TUNEL staining revealed that Arctiin treatment effectively attenuated DOX-induced overproduction of reactive oxygen species (ROS), reduced apoptosis, and improved cell viability (Figures 5J–L).

To determine whether Arctiin interferes with the anti-tumor efficacy of DOX, we performed CCK-8 assays in B16 melanoma cells. The results showed that Arctiin did not attenuate DOX-induced cytotoxicity, indicating that its cardioprotective effect does not compromise DOX’s anti-tumor activity (Supplementary Figure 1).

### SIRT1 confers cardiac protection against doxorubicin challenge

**FIGURE 6:**
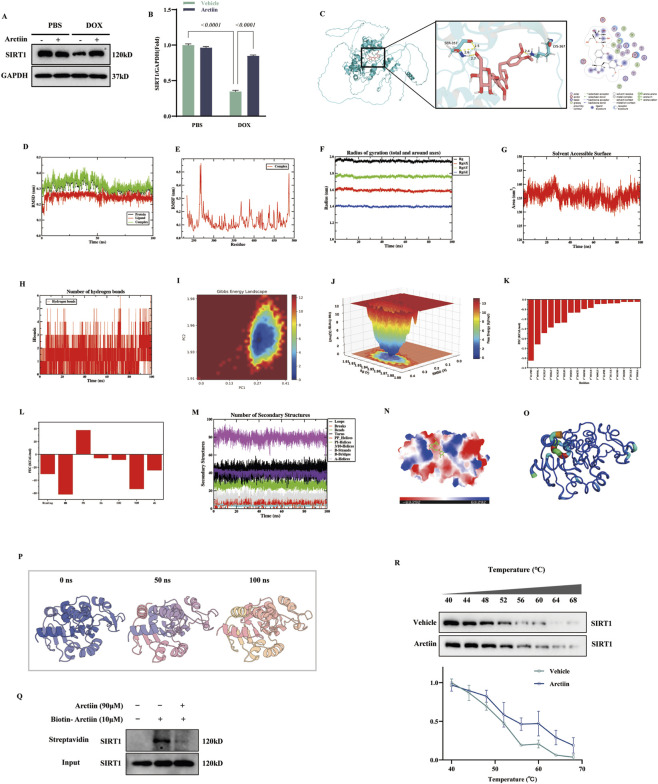
Arctiin could directly bind to and stabilize SIRT1. **(A,B)** Representative Western blot and quantification of SIRT1 in cells treated with vehicle or Arctiin under PBS or DOX conditions (n-3). **(C)** Predicted binding mode of Arctiin in SIRT1. **(D)** Backbone RMSD of protein, ligand and complex. **(E)** Per-residue RMSF of the complex. **(F)** Radius of gyration (total and along X/Y/Z-axes). **(G)** Time evolution of solvent accessible surface area (SASA). **(H)** Number of protein–ligand hydrogen bonds over time. **(I)** 2D Gibbs free-energy landscape along PC1 and PC2. **(J)** 3D free-energy surface as a function of RMSD and Rg. **(K)** Per-residue free-energy contributions identifying key hot-spot residues. **(L)** Decomposition of binding free energy into MM, VDW, PB, SA, COU and total ΔG terms. **(M)** Time evolution of SIRT1 secondary-structure elements during the MD simulation. **(N)** Electrostatic potential surface (APBS; red, negative; blue, positive) highlighting the Arctiin-binding groove. **(O)** SIRT1 structure colored by B-factor/RMSF (blue to red from low to high flexibility). **(P)** Representative snapshots of the Arctiin–SIRT1 complex at 0, 50 and 100 ns. **(Q)** SIRT1 was incubated with biotin-Arctiin (10 μM) with or without unlabeled Arctiin (90 μM), followed by streptavidin pull-down and Western blot. Unlabeled Arctiin competes for SIRT1 binding (n = 3). **(R)** CETSA of SIRT1 in cells treated with vehicle or Arctiin. Data were presented as mean ±S.D.

SIRT1 is a star target of drug screening, which is involved in various physiological processes including anti-inflammation and anti-oxidation (Wang et al., 2021). Our previous work indeed found that SIRT1 activation and increased expression play a protective role in DOX-induced cardiotoxicity (Yuan et al., 2018). In this study, we first examined the protein level of SIRT1 to clarify the regulatory effect of Arctiin. The results showed that, compared with the normal control group, doxorubicin (DOX) treatment significantly downregulated SIRT1 protein expression, while Arctiin effectively reversed the DOX-induced suppression of SIRT1 expression and restored its protein levels (Figures 6A,B). Based on these findings, we hypothesized that Arctiin might exert its effect through direct regulation of SIRT1. To investigate whether direct binding occurs, molecular docking analysis was further performed. The docking results revealed that Arctiin stably occupies the conserved active pocket inside the SIRT1 protein and forms a stable interaction network with key residues. Specifically, hydrogen bonds (2.4–2.7 Å) were formed between the ligand and SER-357, LYS-367, and GLU-402. In addition, hydrophobic packing with residues such as PHE-358, LEU-410, and VAL-404 enhanced binding stability. The presence of both acidic and basic residues within the pocket conferred distinct polar and charge-complementary characteristics to the binding site (Figure 6C).

To verify the binding stability predicted by docking and to elucidate the conformational changes and interaction mechanisms after Arctiin binds to SIRT1, a 100 ns molecular dynamics simulation was conducted. System stability analysis showed that the root-mean-square deviation of Cα atoms for the complex, free protein, and ligand increased slightly during the initial 10–15 ns and then entered a plateau phase with relatively small fluctuations, remaining within a range of approximately 0.15–0.40 nm. The RMSD of the complex was slightly higher than that of the free protein and the ligand, indicating that ligand binding induced local conformational adjustments without causing significant shifts in the backbone structure, suggesting that the system remained stable throughout the simulation (Figure 6D). Per-residue root-mean-square fluctuation analysis indicated that most residues of SIRT1 had RMSF values below 0.3 nm, and the RMSF values of residues around the binding pocket were significantly lower than those in the unbound group, demonstrating that Arctiin binding specifically enhanced the structural rigidity of the active center region (Figure 6E). Further analysis of the radius of gyration and solvent-accessible surface area showed that the Rg value of the complex remained between 2.15 and 2.20 nm throughout the simulation, which was lower and exhibited smoother fluctuations compared to the unbound group. The SASA value remained stable within 130–145 nm^2^, suggesting that Arctiin binding made the spatial structure of SIRT1 more compact (Figures 6F,G). Regarding interactions, hydrogen bond analysis revealed that the complex consistently maintained approximately 1–3 stable hydrogen bonds throughout the simulation, occasionally increasing briefly to 4–5, and never dissociated completely. This indicates that Arctiin maintained an effective hydrogen-bond network within the binding pocket, providing crucial support for its relatively high binding affinity (Figure 6H). Free energy landscape analysis showed concentrated and deep low-energy regions in both 2D and 3D plots, indicating that the Arctiin-SIRT1 complex was confined to a stable energy potential well with limited conformational fluctuations, consistent with the highly stable state reflected by indicators such as RMSD and Rg (Figures 6I,J). Binding free energy decomposition and residue contribution analysis demonstrated that the total binding free energy was markedly negative, suggesting that the binding process is thermodynamically spontaneous and favorable, with van der Waals interactions serving as the primary driving force. Key residues such as PRO-411, VAL-404, GLU-412, and GLY-356 contributed most significantly to binding, most of which are located inside or near the entrance of the pocket and represent critical “hotspot” residues (Figures 6K,L). Additionally, secondary structure analysis showed that the overall secondary structure of the protein remained stable during the simulation, with no large-scale unfolding observed (Figure 6M). Electrostatic potential surface and B-factor analysis further revealed that the binding pocket exhibits an electrostatic potential distribution complementary to the ligand, and this region displayed lower thermal motion and higher rigidity (Figures 6N,O). Conformational trajectory comparison indicated that, although minor local adjustments occurred, the overall protein folding pattern and the ligand binding pose remained largely consistent throughout the simulation (Figure 6P).

To experimentally validate the direct interaction between Arctiin and SIRT1, we performed a biotin-labeled Arctiin pull-down assay. SIRT1 protein was incubated with biotin-labeled Arctiin (10 μM) in the presence or absence of excess unlabeled Arctiin (90 μM), followed by enrichment with streptavidin magnetic beads. Western blot analysis of the pull-down products showed that biotin-Arctiin successfully pulled down SIRT1, indicating a direct interaction. This binding was markedly diminished in the presence of excess unlabeled Arctiin, demonstrating that Arctiin binds to SIRT1 in a specific and competitive manner (Figure 6Q).

Furthermore, a cellular thermal shift assay was performed to validate the binding between Arctiin and SIRT1 at the cellular level. The results showed that as the temperature increased, the intensity of the SIRT1 protein band in the Arctiin-treated group was significantly higher than that in the control group, indicating that Arctiin markedly enhanced the thermal stability of SIRT1 (Figure 6R).

### Arctiin targets the SMURF2-SIRT1 interaction to inhibit ubiquitin-proteasomal degradation

**FIGURE 7:**
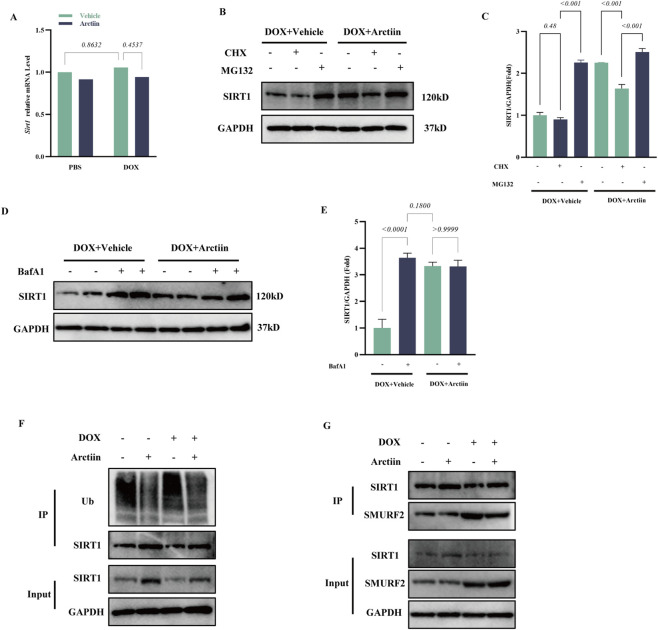
Arctiin inhibits SMURF2-mediated ubiquitination and degradation of SIRT1 **(A)** mRNA level of Sirt1 in DOX or Arctiin treated H9C2 cell (n = 3). **(B)** Western blots and **(C)** statistical result of SIRT1 in H9C2 cells pretreated with arctiin or vehicle, exposed to doxorubicin (DOX), and treated with cycloheximide or MG-132 (n = 3). **(D)** Western blots and **(E)** statistical result of SIRT1 in H9C2 cells pretreated with arctiin or vehicle, exposed to DOX, and treated with bafilomycin A1 (n = 3). **(F)** Co-IP assay and Western blot analysis of SIRT1 ubiquitination levels in H9C2 cells (n = 3). **(G)** Co-IP assay and Western blot analysis of the interaction between SIRT1 and SMURF2 in H9C2 cells (n = 3). Data were presented as mean ±S.D.

Next, we investigated the potential mechanism by which Arctiin regulates SIRT1 protein levels. Since the transcriptional level of SIRT1 remained largely unchanged, while its protein level decreased upon DOX stimulation and was restored following Arctiin treatment (Figures 7A, 6A,B), we hypothesized that Arctiin might regulate SIRT1 stability through post-translational modifications. Existing literature indicates that ubiquitination-mediated proteasomal degradation is a key pathway for SIRT1 downregulation under pathological conditions (Zeng et al., 2025; Gao et al., 2025). To test this, we first examined the degradation kinetics of SIRT1 using the protein synthesis inhibitor CHX. We found that Arctiin pretreatment significantly delayed the degradation of SIRT1 protein in DOX-injured H9C2 cells (Figures 7B,C). To determine whether this stabilizing effect depended on the proteasomal pathway, we concurrently treated cells with the proteasome inhibitor MG-132. The results showed that MG-132 significantly restored the DOX-induced loss of SIRT1 protein, to an extent comparable to the effect of Arctiin (Figures 7B,C), suggesting that Arctiin likely maintains SIRT1 stability by interfering with the proteasomal pathway.

To rule out the involvement of the lysosomal pathway, we further treated cells with the lysosomal inhibitor bafilomycin A1 (BafA1) (Figures 7D,E). The results indicated that Arctiin’s ability to elevate SIRT1 protein levels was unaffected regardless of lysosomal inhibition, confirming that its regulatory action specifically targets the proteasomal degradation pathway.

Subsequently, we examined the effect of Arctiin on SIRT1 ubiquitination. Co-immunoprecipitation assays revealed that DOX treatment significantly enhanced the polyubiquitination modification of SIRT1, whereas Arctiin pretreatment effectively inhibited this modification (Figure 7F). It is known that the E3 ubiquitin ligase SMURF2 is a key mediator of SIRT1 ubiquitination (Yu et al., 2020). To elucidate the specific mechanism by which Arctiin inhibits ubiquitination, we investigated the interaction between SIRT1 and SMURF2. The results demonstrated that DOX promoted the endogenous binding between SIRT1 and SMURF2, while Arctiin treatment markedly weakened this interaction (Figure 7G).

In summary, these findings demonstrate that in DOX-injured cardiomyocytes, Arctiin inhibits the ubiquitination modification of SIRT1 by disrupting its binding to the E3 ubiquitin ligase SMURF2, thereby specifically blocking its degradation via the proteasomal pathway and ultimately stabilizing and upregulating SIRT1 protein levels.

### Essentiality of SIRT1 for the cardioprotective effects of arctiin

**FIGURE 8:**
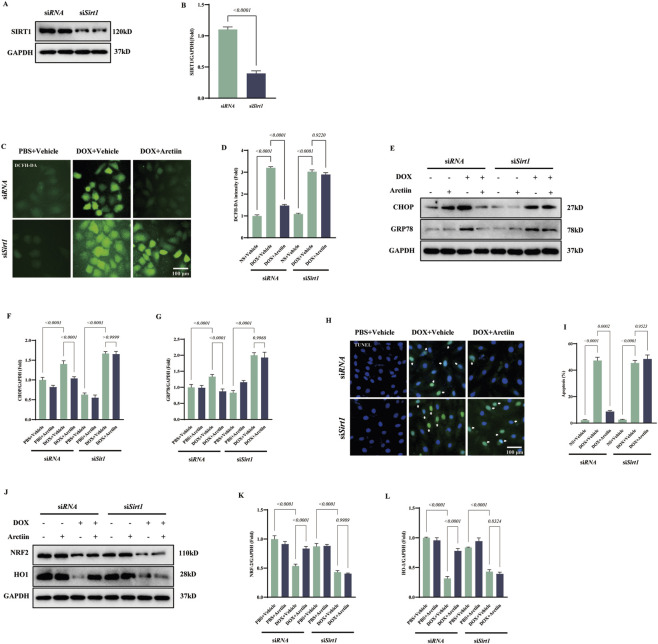
SIRT1 was responsible for Arctiin-mediated beneficial effect in DOX insult. **(A)** Western blot image of SIRT1 in H9C2 cells and **(B)** statistical result (n = 3). **(C,D)** Represent DCFH-DA staining and quantitative statistical result (n = 3). **(E)** Western blot images of endoplasmic reticulum stress-related proteins and **(F,G)** statistical result in H9C2 cells (n = 3). **(H,I)** Represent TUNEL staining and quantitative statistical result in H9C2 cells (n = 3). **(J–L)** Western blot images of downstream signaling proteins of SIRT-1 in H9C2 cells and their statistical results (n = 3). White arrows represent TUNEL-positive cells. Data were presented as mean ±S.D.

To determine whether SIRT1 is required for the protective effects of Arctiin against DOX-induced cardiotoxicity, we first knocked down the SIRT1 in H9C2 cells by transfecting si*Sirt1*. And we verified the knockdown efficiency through Western blot (Figures 8A,B). SIRT1 deficiency abrogated the antioxidant effect of Arctiin, as shown by the restored ROS generation (Figures 8C,D). Similarly, the ability of Arctiin to attenuate endoplasmic reticulum (ER) stress, indicated by the reduced levels of GRP78 and CHOP, was lost upon SIRT1 knockdown (Figures 8E–G). Consistently, the anti-apoptotic effect of Arctiin was reversed, as evidenced by TUNEL assay (Figures 8H,I). Furthermore, SIRT1 knockdown abolished the Arctiin-induced upregulation of its downstream effectors, NRF2 and HO-1 (Figures 8J–L). Collectively, these results demonstrate that SIRT1 is essential for Arctiin to exert its multifaceted protective effects against DOX-induced cardiotoxicity.

**FIGURE 9:**
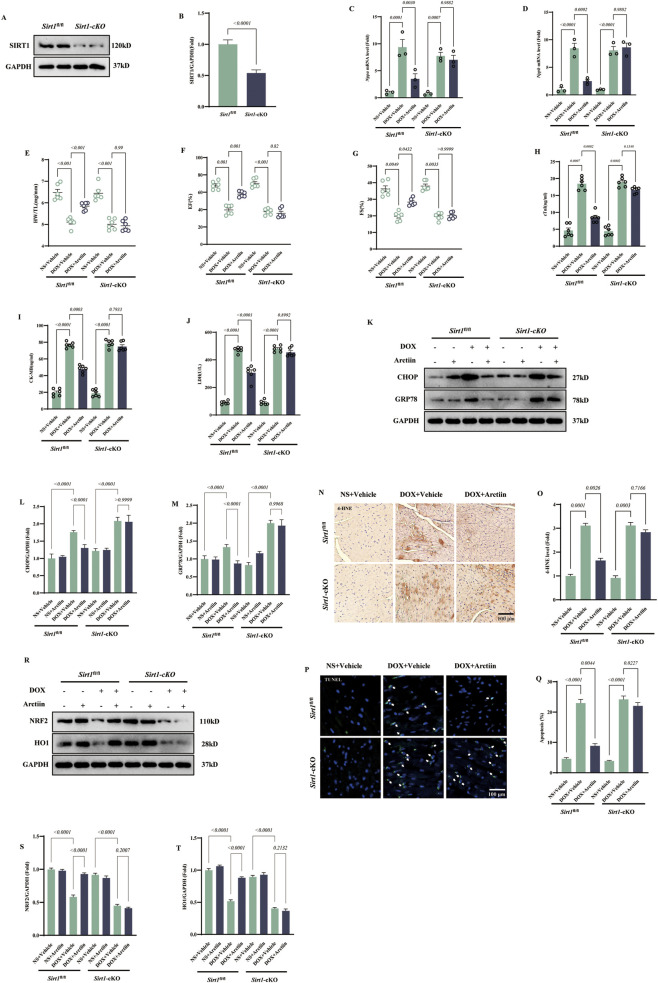
Knockout SIRT1 in cardiomyocytes blunted the beneficial effect of Arctiin in mice. **(A)** Western blot images and **(B)** statistical results of SIRT-1 in mice with different gene types (n = 3). **(C,D)** mRNA level of *Nppa* and *Nppb* of six groups (n = 3). **(E–G)** The ratio of HW/TL, EF, and FS of six groups (n = 6). **(H–J)** Contents of cTnI, CK-MB, and LDH in serum of six groups (n = 6). **(K–M)** Western blot images of endoplasmic reticulum stress-related proteins and partial statistical results in mice (n = 3). **(N)** 4-HNE staining and **(P)** TUNEL staining and **(O,Q)** statistical results (n = 3). **(R–T)** Western blot images of downstream signaling proteins of SIRT1 in mice and their statistical results (n = 3). White arrows represent TUNEL-positive cells. Data were presented as mean ±S.D.

To further validate *in vivo* whether Arctiin exerts cardioprotection via the SIRT1/NRF2 pathway, we employed cardiomyocyte-specific *Sirt1* knockout mice (Figures 9A,B). Consistent with *in vitro* findings, SIRT1 deficiency markedly attenuated the protective effect of Arctiin against DOX-induced cardiac injury, as evidenced by deteriorated cardiac function and structure, along with elevated cardiac injury biomarkers (Figures 9C–J). Mechanistically, in the absence of SIRT1, Arctiin failed to suppress DOX-induced endoplasmic reticulum (ER) stress (Figures 9K–M), and its abilities to inhibit ROS generation and alleviate cardiomyocyte apoptosis were abolished (Figures 9N–Q). Direct examination of key pathway proteins revealed that the Arctiin-induced upregulation of NRF2 and its downstream target HO-1 was completely abolished in cKO mice (Figures 9R–T). These results demonstrate that the protective effects of Arctiin against oxidative stress, ER stress, and apoptosis depend on the activation of the SIRT1/NRF2 pathway.

**FIGURE 10:**
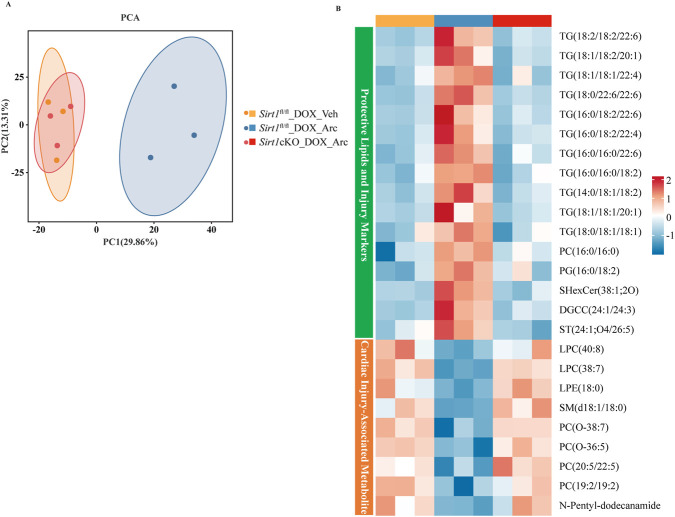
SIRT1-dependent lipid metabolic remodeling by Arctiin after DOX injury. **(A)** PCA score plot shows distinct clustering of the indicated groups, reflecting divergent lipid profiles (n = 3). **(B)** Heatmap analysis indicates that the protective lipid remodeling by Arctiin requires SIRT1, as it is lost in *Sirt1*-cKO mice (n = 3).

To elucidate from a metabolic perspective whether Arctiin exerts its protection by regulating lipid homeostasis via SIRT1, we performed untargeted lipidomics analysis. Principal component analysis (PCA) revealed a clear separation between the Vehicle and Arctiin groups, indicating that Arctiin intervention significantly altered the global lipid metabolic profile under DOX injury (Figure 10A). Heatmap analysis further demonstrated that DOX treatment induced a lipid disorder characterized by upregulation of injury-associated metabolites and downregulation of protective lipids. This dysregulation was significantly reversed by Arctiin (Figure 10B). To identify key lipid classes with relatively high abundance that are modulated by Arctiin, we performed a focused analysis of our lipidomics dataset. The results showed that DOX treatment markedly increased the abundance of multiple ceramide species, while Arctiin treatment effectively reversed this pathological change, restoring their levels toward control values. Conversely, DOX suppressed the levels of glycerophosphoethanolamines, which are critical for membrane integrity, and Arctiin treatment restored these protective lipids. These effects were abrogated upon SIRT1 knockdown (Supplementary Figure 2). Furthermore, this beneficial regulatory effect was completely absent in Sirt1cKO mice, indicating that SIRT1 is essential for Arctiin to ameliorate DOX-induced lipid metabolic disturbances.

In summary, lipid metabolic dysregulation represents a core pathological feature of DOX-induced cardiomyopathy, and Arctiin ameliorates myocardial injury by improving lipid metabolism in a SIRT1-dependent manner.

## Discussion

DOX is widely used in tumor treatment, but its cardiotoxicity cannot be ignored. How to prevent cancer patients from becoming tomorrow’s heart patient deserves attention and research. Dexrazoxane is only one approved drug by Food and Drug Administration (FDA) for the treatment of DOX-induced cardiotoxicity, but the clinical usage is restricted due to its side effect of carcinogenic potential with the high risk of acute myeloid leukemia and myelodysplastic syndrome (Kalyanaraman, 2020; Shaikh et al., 2016). Previous studies demonstrated that many natural compounds have anti-inflammatory and antioxidant properties (Yarmohammadi et al., 2021). Arctiin also has been demonstrated to ameliorate the condition of intracellular ROS generation induced by H_2_O_2_ (Bae et al., 2014). In this study, we demonstrated that Arctiin improves cardiac function in mice and alleviates DOX-induced oxidative stress, ER stress, and apoptosis both *in vivo* and *in vitro*. Furthermore, we found that the protective effect of Arctiin is mediated through the SIRT1/NRF2 pathway. Notably, Arctiin appears to improve lipid metabolism by activating SIRT1, thereby reducing DOX-induced lipotoxicity and further mitigating cardiac injury. Importantly, this protective effect was abolished upon SIRT1 knockdown, confirming the essential role of SIRT1 in the cardioprotective mechanism of Arctiin.

It was proved that oxidative stress is the principle mechanism in DOX-induced cardiotoxicity, ROS are present in cells due to the imbalance of reactive oxygen products in the antioxidant system. Many previous studies demonstrated that excessive production of ROS stimulated lipid peroxidation, calcium dysregulation, and barrier of energy transfer eventually bring about heart failure (Wenningmann et al., 2019). Liu et al. observed that Arctiin could partly increase and stablilize the mitochondrial membrane potential, which in turn helps to reduce the production of ROS (Liu et al., 2021). In this study, we exactly discovered that Arctiin could alleviate DOX-induced oxidative stress in cardiomyocytes. Notably, Chen et al. previously verified that Arctiin could promote the expression of ROS scavenging enzymes through the NRF2/KEAP1/ARE signaling pathway (Chen et al., 2020). To further look for the underlying mechanism for Arctiin to play the antioxitantive role, we then detected the elevated level of Nrf2 related anti-oxidative mRNA and protein in Arctiin treated group. But its specific upstream mechanism of reducing oxidative stress needs to be elaborated. SIRT1 is related to the regulation of oxidative stress and plays a pivotal role in DOX-induced cardiotoxicity (Wang et al., 2021), and is capable of activating NRF2 directly (Wlodarski et al., 2020). We performed a loss-of-function experiment to test our hypothesis that whether NRF2 activation is dependent on SIRT1. Expectedly, deletion of SIRT1 inhibited the activation of NRF2, and extinguished the anti-oxidative effect of Arctiin. A critical question that follows is how Arctiin itself upregulates SIRT1 protein levels. Our data indicate that Arctiin does not enhance SIRT1 gene transcription but specifically prevents the degradation of SIRT1 protein. Mechanistically, we discovered that Arctiin acts as a molecular stabilizer of SIRT1 by directly binding to it and interfering with its interaction with the E3 ubiquitin ligase SMURF2. This intervention effectively inhibits the DOX-induced polyubiquitination of SIRT1, thereby blocking its subsequent recognition and degradation by the proteasome (as evidenced by CHX and MG132 experiments). This novel post-translational regulatory mechanism underscores the precision of Arctiin in enhancing SIRT1 stability, which serves as the foundational step for activating the downstream protective cascade.

The endoplasmic reticulum, which is responsible for folding and transporting mature protein, is hypersensitive to both intracellular and extracellular stimuli. In various ER stress models, ER stress is linked to oxidative stress, these two stress processes accentuated each other in a positive feed-forward loop, interfered with cellular physiological function, and eventually activated apoptosis signaling (Cao and Kaufman, 2014). Previous studies have confirmed that arctigenin, another active compound of Fructus Arcii, has a strong effect on mitigating ER stress (Salama et al., 2021; Zhang et al., 2019; Gu et al., 2012; Kim et al., 2010). In our study, indeed, we found Arctiin lowered the expression of UPR targeted genes, indicating that Arctiin is an effective ER stress alleviator in DOX-induced cardiomyocyte damages. Excess oxidative stress and ER stress eventually determined the cell fate of apoptosis. Previous studies reported that Arctigenin could ameliorate cell apoptosis (Salama et al., 2021; Zhang et al., 2019; Gu et al., 2012). Meanwhile, we expectedly found the expression of the apoptosis-related genes is reduced in Arctiin treated group as well. Surprisingly, these two active compounds of Fructus Arcii exhibited an efficient pro-apoptosis role in anti-tumor therapy. This opposite bioactive effect may be due to the selective cytotoxicity in tumor cells (Kim et al., 2010). But the specific selection mechanism needs to be clarified.

SIRT1, an NAD+-dependant deacetylase, is pivotal in involving homeostasis regulation of oxidative stress, ER stress, apoptosis, inflammatory response, fibrosis, and other physiology or pathology processes (Wang et al., 2021). SIRT1 could alleviate stress by modulating the functions of FOXO, p53, and NF-κB signalings (Salminen and Kaarniranta, 2012). NRF2 regulates a variety of antioxidant proteins, such as HO1, SOD, GSH, CAT, etc. Wang et al. reported that stimulating SIRT1/NRF2 signaling pathway could protect cardiac from apoptosis and endoplasmic reticulum stress (Wang et al., 2019). In our previous study, we found that Sirt1 activation and the expression were suppressed in DOX treated cardiac (Yuan et al., 2018). In agreement with this, we obeserved a significant reduction of Sirt1. After we knockdown the expression of SIRT1, we detected the reduced expression level of NRF2, moreover, the antioxidative, anti-ER stress, and anti-apoptosis roles were inhibited both *in vivo* and *in vitro*. Apparently, Arctiin improved cardiac function and ameliorated myocardial cell loss is mediated by SIRT1/NRF2 signaling.

A novel and significant dimension illuminated by our study is SIRT1’s pivotal role in lipid metabolic homeostasis under DOX challenge (Domi and Hoxha, 2025; Tian et al., 2024). Beyond the classic stress pathways, our lipidomic evidence reveals that DOX induces profound lipid dysregulation (Kretzschmar et al., 2021; Arunachalam et al., 2013). Crucially, Arctiin administration reversed this aberrant lipid profile in a manner strictly dependent on SIRT1. Given SIRT1’s established function as a master metabolic regulator, influencing lipid synthesis, oxidation, and associated transcriptional programs, these findings position SIRT1-mediated lipid metabolic reprogramming as a key mechanism of Arctiin’s action. We propose that by activating SIRT1, Arctiin restores lipid homeostasis, thereby reducing lipotoxicity and breaking the vicious cycle that interlinks metabolic disturbance, oxidative stress, and ER stress, ultimately preserving cardiomyocyte viability.

As a key metabolic regulator, SIRT1 modulates the transcriptional activity of multiple transcription factors and coactivators involved in lipid metabolism through deacetylation, participating in fatty acid oxidation, lipogenesis, and cholesterol metabolism, thereby maintaining lipid metabolic homeostasis in tissues and organs (Zhou et al., 2024; Schug and Li, 2011). For instance, in fatty acid oxidation, PGC-1α is one of the major regulators modulated by SIRT1. Generally, deacetylation of PGC-1α by SIRT1 enhances its transcriptional activity. SIRT1 deacetylates the PGC-1α-PPARα complex, thereby inducing target gene expression and mitochondrial biogenesis. Deacetylation of PGC-1α increases its interaction with PPARα, promoting the transcription of fatty acid oxidation genes in cardiomyocytes (Vega et al., 2000); in lipogenesis, SIRT1 inhibits SREBP-1c activity through deacetylation, downregulating its downstream target genes, such as, FAS and HMGR, thereby reducing *de novo* synthesis of fatty acids and triglycerides (Ponugoti et al., 2010); in cholesterol metabolism, SIRT1 activates LXR through deacetylation, upregulating cholesterol efflux transporters ABCA1 and ABCG1, thereby facilitating reverse cholesterol transport and efflux to maintain cholesterol homeostasis (Zhang et al., 2025; Li et al., 2007). Through this multi-level coordinated regulation, SIRT1 effectively maintains lipid metabolic balance in cardiomyocytes, attenuates lipotoxic injury, and exerts cardioprotective effects. However, how SIRT1 specifically regulates lipid metabolism in the heart, particularly in the context of DOX-induced cardiotoxicity, remains to be fully elucidated. The precise downstream targets and regulatory networks through which SIRT1 orchestrates lipidomic remodeling in cardiomyocytes warrant further investigation. Future studies integrating multi-omics approaches (e.g., transcriptomics, proteomics, and acetylomics) with functional validation will be essential to dissect the detailed molecular mechanisms by which SIRT1-mediated lipid metabolic reprogramming contributes to cardioprotection.

Arctiin, like many natural compounds, likely exerts its biological effects through interactions with multiple cellular targets. Our study identifies SIRT1 as a critical mediator of its cardioprotective effects, as evidenced by loss-of-function experiments demonstrating that SIRT1 is necessary for Arctiin-induced protection. However, we cannot exclude potential contributions from other sirtuin family members (e.g., SIRT3, which regulates mitochondrial ROS; or SIRT6, implicated in cardiac homeostasis) or unrelated pathways (Peng et al., 2024; Wang et al., 2023; Wu et al., 2022). The multi-target nature of Arctiin may in fact represent a therapeutic advantage for addressing the complex pathophysiology of DOX-induced cardiotoxicity, which involves oxidative stress, mitochondrial dysfunction, and apoptosis. Future studies exploring additional targets and pathways will further elucidate the full pharmacological profile of Arctiin.

Therefore, our data support a coherent model wherein Arctiin activates SIRT1, which then orchestrates a coordinated defensive network (Kalyanaraman, 2020): direct activation of the NRF2 antioxidant program (Carvalho et al., 2014), restoration of lipid metabolic homeostasis, and (Cardinale et al., 2015) mitigation of ER stress. These parallel processes converge to inhibit the mitochondrial-dependent apoptotic cascade. The complete abolition of all protective effects upon SIRT1 knockout, both *in vitro* and in cardiomyocyte-specific cKO mice, irrefutably solidifies SIRT1’s non-redundant, central role as the linchpin in this cardioprotective network.

Our study has several limitations that point to future research directions. First, while loss-of-function experiments strongly support the necessity of SIRT1, complementary gain-of-function studies would further strengthen the causality and therapeutic concept. Second, we focused on downstream signaling cascades and metabolic phenotypes; a direct examination of the primary target of DOX injury would provide a more complete picture of the initial insult and SIRT1’s potential role in its preservation. Finally, we have established the central role of SIRT1, but the precise temporal and hierarchical relationships between the SIRT1-mediated lipid metabolic reprogramming, NRF2 activation, and ER stress resolution remain to be fully dissected to understand the orchestration of this protective network.

Cancer survivorship is increasingly shadowed by chemotherapy-induced cardiovascular complications. The discovery of adjunctive therapies that protect the heart without compromising oncological efficacy is of paramount importance. Our work identifies Arctiin as a potent natural cardioprotectant whose efficacy is centrally mediated through the activation of SIRT1. By engaging this key regulator, Arctiin counteracts DOX injury through a multi-faceted mechanism encompassing antioxidant defense, ER stress relief, apoptosis inhibition, and lipid metabolic repair. These findings underscore the high therapeutic value of targeting integrative hubs like SIRT1 and provide a strong rationale for the further development of Arctiin or its derivatives as promising candidates for cardioprotective combinational therapy in oncology.

## Materials and methods

### Reagents

DOX (D1515) was obteined from Sigma-Aldrich (St. Louis, MO, USA). Arctiin (CAS NO.20362-31-6) was produced by Shanghai Winherb Medical Co. (Shanghai, China). The BCA protein assay kit was produced by Pierce (Rockford, IL, USA). Cycloheximide (HY-12320), MG132 (HY-13259), and Bafilomycin A1 (HY-100558) were obtained from MCE (Shanghai, China). Primary antibodies including anti-CHOP (2895), anti-p53 (2524), anti-BAX (2772), anti-BCL2 (2870) and anti-GAPDH (2118) were all purchased from Cell Signaling Technology (Boston, MA, USA). Primary antibodies including anti-SOD1 (ab16831), anti-SOD2/MnSOD (ab68155), anti-GRP78 (ab21685) were purchased from Abcam (Cambridge, UK). Anti-XBP1(A1731), anti-phosph-eIF2α(AP0692), anti-Caspase12, anti-SIRT1, anti-NRF2 were obtained from Abclonal Technology Company (Wuhan, China). Anti-ATF6b (15794-1-AP) was produced by Proteintech Group (Wuhan, China). DAB Detection Kit was produced by GeneTech Company (Shanghai, China). Dihydroethidium (DHE, S0063), Reactive Oxygen Species Assay Kit (S0033S), and One Step TUNEL Apoptosis Assay Kit (C1088) was produced by Beyotime Company (Shanghai, China). NADPH oxidase assay kit, total SOD assay kit, Malondialdehyde (MDA) assay kit, and Caspase-3 activity assay kit were all generated by Nanjing Jiancheng Bioengineering Institute (Nanjing, China). GSH and CAT assay kit were produced by Shanghai Enzyme-linked Biotechnology Co. (Shanghai, China). Sangon (Shanghai, China) generated the small interfering RNA against *Sirt1* (si*Sirt1*) and corresponding negative control.

### Animals

The animal care and experimental procedures were conducted in compliance with the Guidelines for the Care and Use of Laboratory Animals published by the United States National Institutes of Health (NIH Publication, revised 2011), following approval from the Animal Care and Use Committee of Renmin Hospital, Wuhan University (Wuhan, China). The blind methods were used in all animal treatments and subsequent analyses. Male C57BL/6 mice (8–10 weeks old, 23.5 g–27.5 g) were bred and sold by the Institute of Laboratory Animal Science, Chinese Academy of Medical Sciences (Beijing, China). It is required that all animals be kept in specific pathogen free environments (SPF) with regular 12/12 h cycles of light/dark, which could maintain a constant temperature of 20 °C–25 °C and a suitable humidity level of 50%–75%. After 1 week adaptive feeding, these mice were randomly divided into 4 groups, including Normal Saline (NS)+Vehicle (Veh), NS+Arctiin, DOX+Veh, DOX+Arctiin. The mice in DOX groups were gotten one single intraperitoneally inject of DOX (15 mg/kg) to induce acute cardiac injuries, the control mice were given same volumes of normal saline. The mice in Arctiin groups were gotten intragastrical administration of Arctiin (80 mg/kg/day) consecutive 7 days simultaneously from the day of injection of DOX. The dosage of Arctiin used *in vivo* was selected based on our previous studies demonstrating optimal cardioprotective efficacy at this concentration (Li et al., 2017). Besides, to explore the role of SIRT1, *Sirt1* conditional floxed mice (Generated by Nanjing University) were hybridized with mice carrying the *α-Mhc-MerCreMer* transgene (Purchased from Jackson Laboratory) to constructed cardiac-conditional *Sirt1* knock out (*Sirt1*-cKO) mice as our previous study (Hu et al., 2020).

### Echocardiography and hemodynamic detection

Transthoracic echocardiography and invasion hemodynamic monitoring were performed as the methods we used in previous studies (Yuan et al., 2018). We anesthetized the mice before ultrasound, 2% isoflurane were used to induce anesthesia and maintained anesthesia in 1.5% isoflurane. Transthoracic echocardiography were tested through a Vevo 3100 ultrasound (FUJIFILM, VisualSonics) to record cardiac morphological and functional data of a heart averaged from three to six cardiac cycles.

### Cell culture and treatments

H9C2 cells were obtained from the Cell Bank of the Chinese Academy of Sciences (Shanghai, China). Dulbecco’s Modified Eagle’s Medium (DMEM, GIBCO) with 10% Fetal Bovine Serum (FBS, GIBCO) were used to culture the cells. Next, cells were given serum-free medium for 16 h for cell cycle synchronizing. Then, cells were randomly incubated with or without Arctiin (100 μM) in the presence or absence of DOX (1 μM) for 24 h (Li et al., 2017). The concentration of Arctiin (100 µM) used for *in vitro* experiments was selected based on our previous dose-response studies demonstrating optimal efficacy at this concentration in cardiomyocytes (Li et al., 2017). To explore the role of SIRT1, we conducted cells transfection experiments with si*Sirt1* or si*RNA* for 24 h by Lipo6000™ transfection reagent (Beyotime, Shanghai). Based on experimental requirements, cells were stimulated with either Cycloheximide (100 μg/mL, 12 h), MG132 (20 μM, 8 h), or Bafilomycin A1 (0.1 μM, 4 h). Each experiment was performed in triplicate biological replicates. All procedures were according with our previous study (Yuan et al., 2018).

To evaluate whether Arctiin interferes with the anti-tumor efficacy of DOX, B16 mouse melanoma cells (obtained from the Cell Bank of the Chinese Academy of Sciences, Shanghai, China) were cultured in DMEM supplemented with 10% FBS. Cells were seeded into 96-well plates at a density of 5 × 10^3^ cells per well and incubated overnight. Subsequently, cells were treated with DOX (1 μM) in the presence or absence of Arctiin (100 μM) for 24 h. Then, 10 μL of CCK-8 solution (Beyotime, #C0037) was added to each well and incubated for an additional 2 h at 37 °C. Absorbance was measured at 450 nm using a microplate reader. Cell viability was calculated as the percentage relative to untreated control cells.

### Molecular biological detection

Western blot and quantitative real-time PCR were operated as methods we used to follow (Ma et al., 2017). In brief, total proteins were extracted from mice left ventricles and cultured cells. Then, these proteins were electrophoretically separated in 10% SDS-PAGE and then electrophoretically transferred to PVDF membranes. Next, these proteins were incubated with targeted primary antibodies at 4 °C over nights, and with corresponding secondary antibodies at room temperature for 1 h.

Total RNA was extracted by TRIzol reagent and subjected reverse transcription for cDNA. Quantitative real-time PCR was conducted using SYBR GREEN I. *Gapdh* was chosen as a reference.

### TUNEL staining

The TdT-mediated dUTP Nick-End Labeling (TUNEL) Staining procedure was as previously described (Yuan et al., 2018). One Step TUNEL Apoptosis Assay Kit was used to detect positive condition of TUNEL. Then, the stained samples were sealed with DAPI, and were observed and recorded using the fluorescence microscope (OLYMPUS, Tokyo, Japan).

### Measurement of intracellular ROS

The condition of intracellular ROS was evaluated by DCFH-DA probe for H9C2 cells and DHE probe for heart tissues as we previously described (Hu et al., 2020). The living cells were cultured in serum-free medium containing DCFH-DA stain (5 μM) for 1 h and washed with PBS 3 times under dark conditions. After thawing at room temperature and washing with PBS 3 times, frozen hearts slices were incubated at 37 °C for 30 min using DHE (5 μM) and washed with PBS 5 times. Then, all slices and cells datas were gathered using the OLYMPUS DX51 fluorescence microscope (Tokyo, Japan).

### Biochemical analysis

The level of serum biomarkers of myocardial injury, such as, CK-MB (creatine kinase isoenzymes), cTnI (cardiac isoform of Tropnin I), and LDH (lactate dehydrogenase) were detected by an automatic biochemical analyzer (ADVIAs 2400, SiemensLtd., China) as previously described (Hu et al., 2020). Measurement of total activities of NADPH oxidase, CAT, SOD, and Caspase3, and the level of MDA and GSH were finished through the commercially available ELISA kits.

### Molecular dynamics (MD) simulations

MD simulations of the protein-ligand complexes were performed using the GROMACS 2025.3 software package (Pall et al., 2020). The system was solvated in a cubic periodic boundary box using the TIP3P water model and the AMBER14SB force field (Maier et al., 2015). Atomic partial charges and the optimized molecular geometry for the ligands were obtained from quantum mechanical (QM) calculations conducted with the ORCA program (version 6.0) (Neese, 2025). The QM calculations proceeded in two stages: 1) Geometry optimization of the molecular structure in the gas phase using the composite B97-3c density functional theory (DFT) method; 2) A subsequent, more precise single-point energy calculation on the optimized geometry to determine the electronic distribution, employing the B3LYP functional with D3 dispersion correction and the def2-TZVP basis set. The RIJCOSX approximation was utilized to accelerate calculations while maintaining accuracy. The molecular electrostatic potential (ESP) derived from the single-point calculation was used to generate restrained electrostatic potential (RESP) charges via the Multiwfn program (Lu, 2024). Topology files for the small molecules were generated using the sobtop software, and ions were added to achieve a physiological salt concentration (150 mM NaCl).

Following energy minimization and a stepwise NPT equilibration (100 ps) with positional restraints, a 100 ns production simulation was conducted using an integration time step of 2 fs. Trajectories were processed to remove periodic boundary artifacts and to center the system. Analyses performed included: the root-mean-square deviation (RMSD) and radius of gyration (Rg) of the protein backbone Cα atoms, per-residue root-mean-square fluctuation (RMSF), solvent-accessible surface area (SASA), and the number of protein-ligand hydrogen bonds.

The binding free energy (ΔGbind) was calculated using the molecular mechanics Poisson–Boltzmann surface area (MM-PBSA) method. This efficient post-processing framework is suitable for evaluating the binding affinity of protein-ligand complexes. The method quantifies the total binding free energy and decomposes it into key components: the molecular mechanics interaction energy (ΔEMM), comprising van der Waals (ΔEvdW) and electrostatic (ΔEele) contributions, and the solvation free energy, which includes the polar solvation term (ΔGPB) and the non-polar solvation term (ΔGSA). Although MM-PBSA is less rigorous than methods such as free energy perturbation (FEP) or thermodynamic integration (TI), its lower computational cost makes it suitable for distinguishing relative binding strengths and analyzing molecular recognition in large systems.

To gain deeper insight into the binding mechanism, a per-residue free energy decomposition analysis was performed. This analysis quantitatively allocates the total binding free energy to individual amino acid residues of the protein receptor, calculating the changes in van der Waals energy, electrostatic interaction energy, and solvation energy between each residue and the ligand. This identifies key “hotspot” residues within the binding pocket, providing molecular-level insights for understanding binding specificity and guiding structural optimization.

We further conducted molecular electrostatic potential (ESP) analysis to visualize charge distribution and elucidate the electrostatic environment of the molecules. Additionally, two-dimensional (2D) and three-dimensional (3D) free energy landscape (FEL) plots were constructed to visualize the conformational stability of the complexes. These landscapes, using the radius of gyration (Rg) and root-mean-square deviation (RMSD) as reaction coordinates, illustrate the free energy distribution of the system across conformational space. Deep blue basins indicate global free energy minima, corresponding to the most thermodynamically stable conformations.

### Biotin-arctiin pull-down assay

To validate the direct binding between Arctiin and SIRT1, a biotin-labeled Arctiin pull-down assay was performed. H9C2 cells were lysed in NP-40 lysis buffer (Vazyme, China) supplemented with complete protease inhibitor cocktail (Roche, Switzerland) on ice for 30 min. After centrifugation, the supernatant was collected and protein concentration was determined using a BCA protein assay kit (Beyotime, China). Streptavidin magnetic beads (Beyotime, P2151) were washed three times with binding buffer (PBS containing 0.1% NP-40 and protease inhibitors) and incubated with biotin-Arctiin (20 μM) for 1 h at room temperature to allow immobilization. After washing to remove unbound biotin-Arctiin, 500 μg of cell lysate was added and incubated overnight at 4 °C with gentle rotation. To assess binding specificity, excess unlabeled Arctiin (80 μM) was simultaneously added to the lysate as a competitive inhibitor. The beads were then washed five times with washing buffer (PBS containing 0.5% NP-40 and protease inhibitors) to remove non-specifically bound proteins. Bound proteins were eluted by boiling the beads directly in 2× SDS loading buffer at 95 °C for 10 min and subjected to Western blot analysis.

### Cellular thermal shift assay (CETSA)-Western blot

The cell samples were cultured for 24 h in the presence of DOX and with either Arctiin or Vehicle control according to their respective groups. Each sample was subsequently divided into 8 aliquots. A PCR instrument was preheated to 40, 44, 48, 52, 56, 60, 64, and 68 °C. The protein samples underwent heat treatment in the PCR instrument for 5 min, after which the heat-treated cells were resuspended in NP40 lysis buffer. Three cycles of freezing in liquid nitrogen and thawing were performed to lyse the cells. The lysates were then centrifuged at 4 °C and 12,000 rpm for 15 min to retain proteins that were intact and non-degraded. Loading buffer was added to the samples, which were then denatured at 95 °C for 5 min. Finally, Western Blot analysis was performed to detect SIRT1 protein expression.

### Immunoprecipitation

Treated H9C2 cells were washed three times with PBS and lysed using NP-40 Lysis Buffer (Vazyme, China). The lysates were centrifuged at 12,000 rpm for 10 min at 4 °C. The resulting supernatant was divided into two aliquots: 40 μL designated as the Input sample and 160 μL as the immunoprecipitation (IP) sample. Protein A/G Magnetic Beads were pre-washed with PBST Washing Buffer and subsequently incubated with the SIRT1 antibody for 30 min at room temperature to facilitate antibody conjugation. Following four washes with PBST using magnetic separation, the beads were resuspended and prepared for the IP procedure. The IP sample was incubated with the antibody-conjugated Protein A/G Magnetic Beads (Vazyme, China) for 30 min at room temperature. After four rounds of washing with PBST and magnetic separation, the supernatant was discarded. The beads were then resuspended in 1× SDS-PAGE Loading Buffer, thoroughly mixed, and heated at 95 °C for 5 min to elute the immunoprecipitated proteins. The supernatant containing the eluted proteins was collected via magnetic separation for subsequent SDS-PAGE analysis.

### Untargeted lipid metabolome profiling

Grinding the samples to powder form by using a grinder. Next, weigh 100 mg of sample powder using an electronic balance (MS105DΜ) and add 1 mL of 70% methanolic aqueous. Ultrasound extraction for 30 min, then store the sample in a refrigerator at 4 °C. After centrifugation (rotationspeed 12500 rpm, 10 min), the supernatant was aspirated, and the sample was filtered through a microporous membrane (0.22 μm pore size) and stored in the injection vial for UPLC-MS/MS analysis.

All samples were acquired by the LC-MS system followed machine orders. The analytical conditions were as follows, UPLC: column, Waters ACQUITY Premier HSS T3 Column 1.8 µm’ 2.1 mm * 100 mm; column temperature, 40 °C; flow rate, 0.30 mL/min; injection volume, 2 μL. The mobile phase was consisted of solvent A, ammonium acetate mixed solution (5 mmol/L), and solvent B, pure acetonitrile.Sample measurements were performed with a gradient program that employed the starting conditions of 95% A, 5% B, Within 10 min, a linear gradient to 5% A, 95% B was programmed, and a composition of 5% A, 95% B was kept for 2 min. Subsequently, a composition of 95% A, 5.0% B was adjusted within 0.1 min and kept for 2.9 min. The flow velocity was set as 0.3 mL per minute; The column oven was set to 40 °C; The injection volume was 2 μL. The effluent was alternatively connected to an LC-MS/MS.

The data acquisition was operated using the data-dependent acquisition (DDA) mode using MSDIAL Software. The source parameters were set as follows: sheath gas flow rate, 320 Arb; aux gas flow rate, 40 Arb; temperature (TEM), 350 °C in positive or negative modes, respectively; and ion spray voltagefloating (ISVF), 3400 V or −3200 V in positive or negative modes, respectively. The TOF MS scan parameters were set as follows: mass range, 85–1250 Da.

### Statistical analysis

All data are presented as mean ± standard deviation (S.D.). Statistical analyses were performed using GraphPad Prism (Version 9.0). For comparisons between two independent groups with normal distribution, an unpaired two-tailed Student's t-test was used if variances were homogeneous. For comparisons among multiple independent groups with normal distribution, One-way or two-way analysis of variance (ANOVA) with Turkey post-hoc analysis were used to analyse the differences in two or multiples groups. P value < 0.05 was determined as statistically significant. All “n” values represent biological replicates (independent animals), unless otherwise stated.

## Data Availability

The original contributions presented in the study are included in the article/Supplementary Material, further inquiries can be directed to the corresponding author.
