# Supplementary material for: Targeting SIRT1 stability: arctiin ameliorates doxorubicin-induced cardiac injury by inhibiting SMURF2 binding and enhancing protective lipid metabolism
Source: Front Pharmacol. 2026 Apr 23;17:1797815. doi: 10.3389/fphar.2026.1797815 (PMC13149282; doi:10.3389/fphar.2026.1797815)
Supplement: Supplementary file 1 [file Supplementaryfile1.docx]

Supplementary Materials

**This file includes:**

Supplementary Figure. 1 to 2


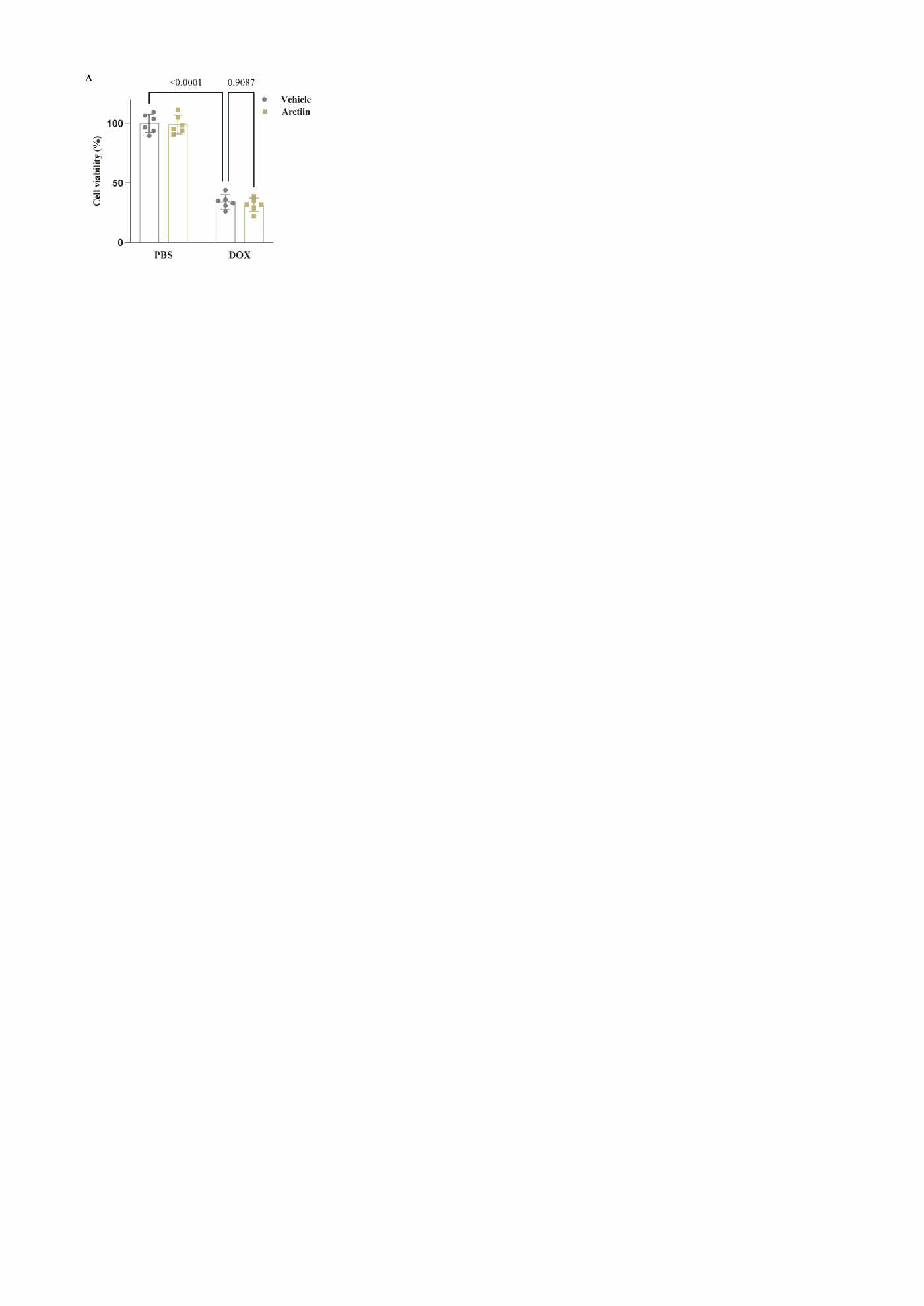


**Supplementary Figure 1. Arctiin does not interfere with DOX's anti-tumor efficacy in B16 melanoma cells.**

(A) B16 cells viability was assessed by CCK-8 assay (n=6). Data were presented as mean ±S.D.


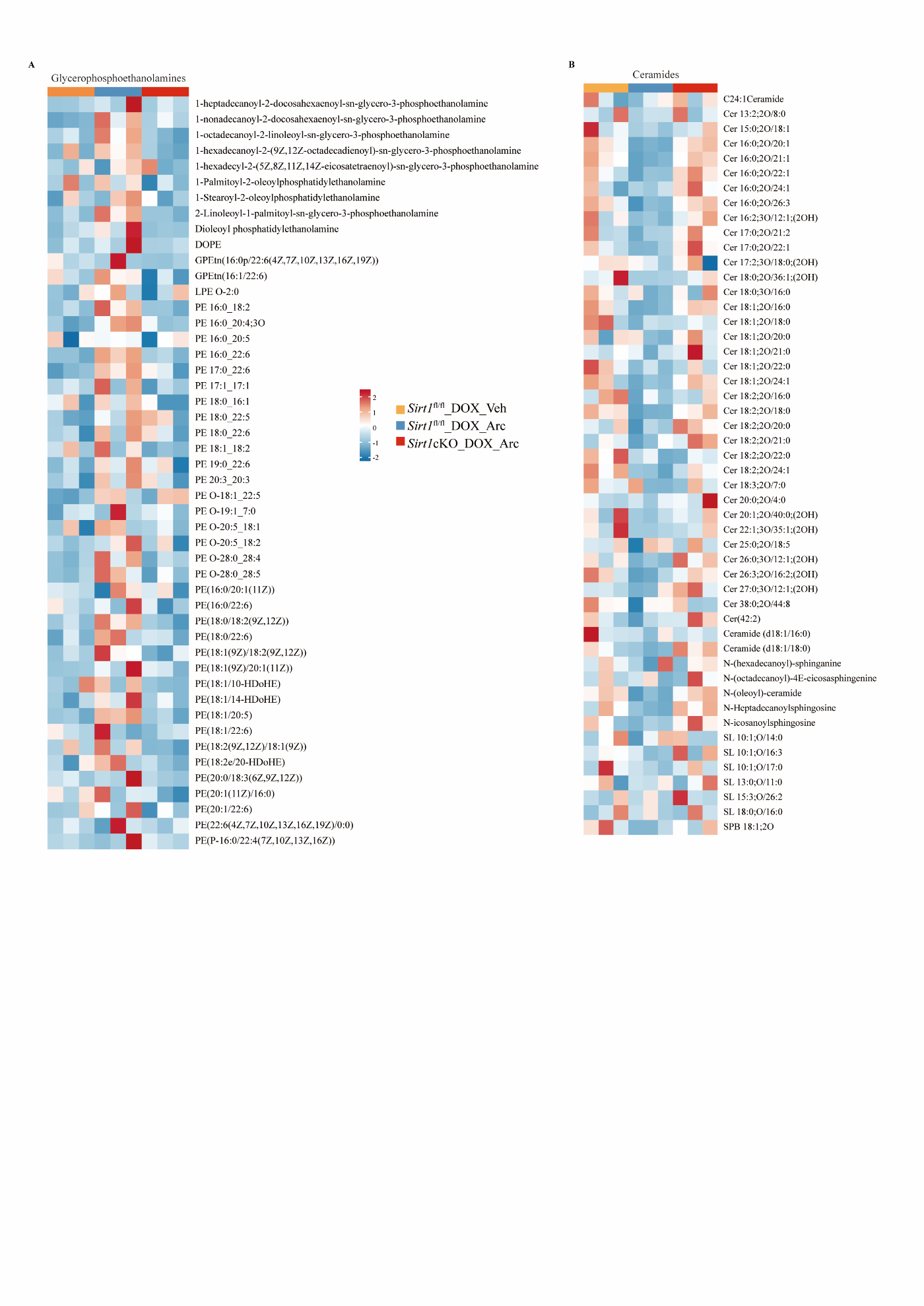


**Supplementary Figure 2. Arctiin reverses DOX-induced dysregulation of glycerophosphoethanolamines and ceramides in a SIRT1-dependent manner.**

(A-B) Heatmap showing the relative abundance of representative glycerophosphoethanolamine (PE) and ceramide species.
